# DEAF1 Binds Unmethylated and Variably Spaced CpG Dinucleotide Motifs

**DOI:** 10.1371/journal.pone.0115908

**Published:** 2014-12-22

**Authors:** Philip J. Jensik, Jesse D. Vargas, Sara N. Reardon, Shivakumar Rajamanickam, Jodi I. Huggenvik, Michael W. Collard

**Affiliations:** Department of Physiology, Southern Illinois University School of Medicine, Carbondale, Illinois, United States of America; University of Bonn, Institut of experimental hematology and transfusion medicine, Germany

## Abstract

DEAF1 is a transcriptional regulator associated with autoimmune and neurological disorders and is known to bind TTCG motifs. To further ascertain preferred DEAF1 DNA ligands, we screened a random oligonucleotide library containing an “anchored” CpG motif. We identified a binding consensus that generally conformed to a repeated TTCGGG motif, with the two invariant CpG dinucleotides separated by 6–11 nucleotides. Alteration of the consensus surrounding the dual CpG dinucleotides, or cytosine methylation of a single CpG half-site, eliminated DEAF1 binding. A sequence within the *Htr1a* promoter that resembles the binding consensus but contains a single CpG motif was confirmed to have low affinity binding with DEAF1. A DEAF1 binding consensus was identified in the *EIF4G3* promoter and ChIP assay showed endogenous DEAF1 was bound to the region. We conclude that DEAF1 preferentially binds variably spaced and unmethylated CpG-containing half-sites when they occur within an appropriate consensus.

## Introduction

Deformed Epidermal Autoregulatory Factor 1 (DEAF1) is a transcription factor that binds to TTCG half-sites through a centralized DNA binding SAND (Sp-100, AIRE, NucP41/75 and DEAF1) domain [1]–[3]. The SAND domain contains a positively charged region encompassing a conserved KDWK motif [3]. An adjacent zinc finger domain and nuclear localization signal are necessary for DEAF1-DNA interactions [4]. Transcriptionally, DEAF1 displays dual activity, repressing its own promoter activity while activating other promoters such as *Eif4g3* [3], [5], [6]. DEAF1-DEAF1 and DEAF1-Ku70 protein interactions also occur through the SAND domain [4], [7]. DEAF1 contains a nuclear export signal that acts as part of a second DEAF1-DEAF1 and DEAF1-LMO4 protein interaction domain [4], [8]–[10]. A C-terminal cysteine rich MYND (Myeloid translocation protein 8, Nervy, and DEAF1) domain likely mediates other protein-protein interactions [11].

Specific mutations in the SAND domain of the *DEAF1* gene result in moderate to severe non-syndromic intellectual disability in humans [6], [12]. These mutations eliminate or greatly reduce both DEAF1 interactions with TTCG-containing DNA sequences and DEAF1 transcriptional repression of its own promoter [6]. DEAF1 is also linked to human mood disorders [13]–[16], cancer [17], [18], autoimmune disorders [5], [19] and interferon-β production [20]. DEAF1 deficiency leads to neural tube closure defects in mice [21] and early embryonic arrest in *Drosophila* [22]. Deletion of *Deaf1* in mouse brain results in an anxiety-like phenotype and causes severe deficits in 24-hour contextual memory [6].

In our previous study, a degenerate random oligonucleotide library was used to identify TTCG motifs in DEAF1-binding sequences [2]. Subsequently, Burnett et al. [23] demonstrated that introduction of an “anchored” CpG half-site core into a degenerate oligonucleotide library allowed identification of the optimal spacing and preferred sequences surrounding the CpG-containing half-sites for the SAND domain-containing glucocorticoid modulatory element binding 1/2 (GMEB1/2) protein. The objectives of this study were to: 1) further delineate the DNA consensus sequence required for DEAF1 binding using affinity selection of a CpG-anchored oligonucleotide library, 2) assess the effects of CpG methylation on DEAF1-DNA interactions, and 3) characterize the binding of DEAF1 to a sequence within the *EIF4G3* promoter. Increased understanding of DNA sequences that DEAF1 can or cannot bind should aid in identifying potential DEAF1 target genes and provide insight into their regulation in normal biology and DEAF1-related disease.

## Materials and Methods

### Plasmids

GST-DEAF1 and DEAF1-FLAG constructs have been previously described [4] and were derived from human DEAF1 cDNA (accession number AF049459).

### Purification of DEAF1 proteins

Full-length recombinant bacterial expressed GST-DEAF1 and HEK293T expressed DEAF1-FLAG proteins were purified as previously described [4], [7]. Relative purities of the proteins are shown in S1 Figure.

### DEAF1 DNA Consensus Selection

DEAF1 affinity selection of DNA sequences was similar to that previously described [2] using GST-DEAF1 and DEAF1-FLAG proteins, but was modified as in [23] to include an anchored CpG dinucleotide in degenerate oligonucleotides and to also include an electrophoretic mobility shift assay (EMSA) for affinity purification of DEAF1-DNA complexes. The degenerate oligonucleotide library was made with the following three oligonucleotides:

63-mer-5′-CTGCTGGATCCTGCAGCTCTGAGN_3_ **CG**N_13_GTCTGACAAGCTTCTAGAGTCA-3′

Selection Forward Primer- 5′-CTGCTGGATCCTGCAGCTCTGAG-3′

Selection Reverse Primer- 5′-TGACTCTAGAAGCTTGTCAGAC-3′

The 63-mer oligonucleotide consists of an 18-mer of random nucleotides with an internal anchored CpG dinucleotide flanked by a 5′ 23-mer with a *Bam*HI site and a 3′ 22-mer with a *Hind*III site (sites are underlined) to facilitate subcloning into pBluescript II KS+ vector. Briefly, GST-DEAF1 fusion protein immobilized on glutathione-agarose beads was incubated with the CpG anchored degenerate oligonucleotide library. Bound oligonucleotides were eluted and amplified by PCR using Selection Forward and Reverse primers and one-tenth of the PCR product was used in the next round of selection. A total of 6 rounds of selection were performed. Oligonucleotides in the final round of selection were amplified by PCR (10 cycles) with ^32^P-ATP to generate radiolabeled oligonucleotides that were used in a single round of EMSA selection with mammalian expressed DEAF1-FLAG protein. DNA in the shifted bands were excised, amplified by PCR and digested with *Hind*III and *Bam*HI prior to subcloning. DNA from individual colonies was sequenced on the CEQ8000 DNA sequencer (Beckman Coulter) using T7 and T3 primers.

### Consensus Analysis

Sequences were compared and aligned using MEME (Multiple Em for Motif Elicitation) [24]. Resultant half-site sequences were further analyzed using D-Matrix [25] and pictogram (http://genes.mit.edu/pictogram.html). Genomic scans were performed using RSA-Tools Genomic Scale PatternSearch [26] from the RSAT server, Brussels, Belgium.

### EMSA Binding Analysis

The indicated ^32^P-Labeled dsDNA probes were synthesized by PCR and incubated with 200 ng of DEAF1-FLAG protein for 30 min at room temperature in 1x EMSA binding buffer with 1 µg of dA:dT. Complexes were separated on 5% native polyacrylamide gels and migration of the DNA probes were visualized by PhosphorImager. EMSA analysis using fluorescent IR700 and IR800 DNA probes for S6con and N52-69 was conducted as previously described [6]. A dsDNA probe for the 5-hydroxytryptamine receptor 1A (*Htr1a*) promoter region is based upon the “mouse#1” sequence described in Fig. 1 of [27] using the primers

**Figure 1:**
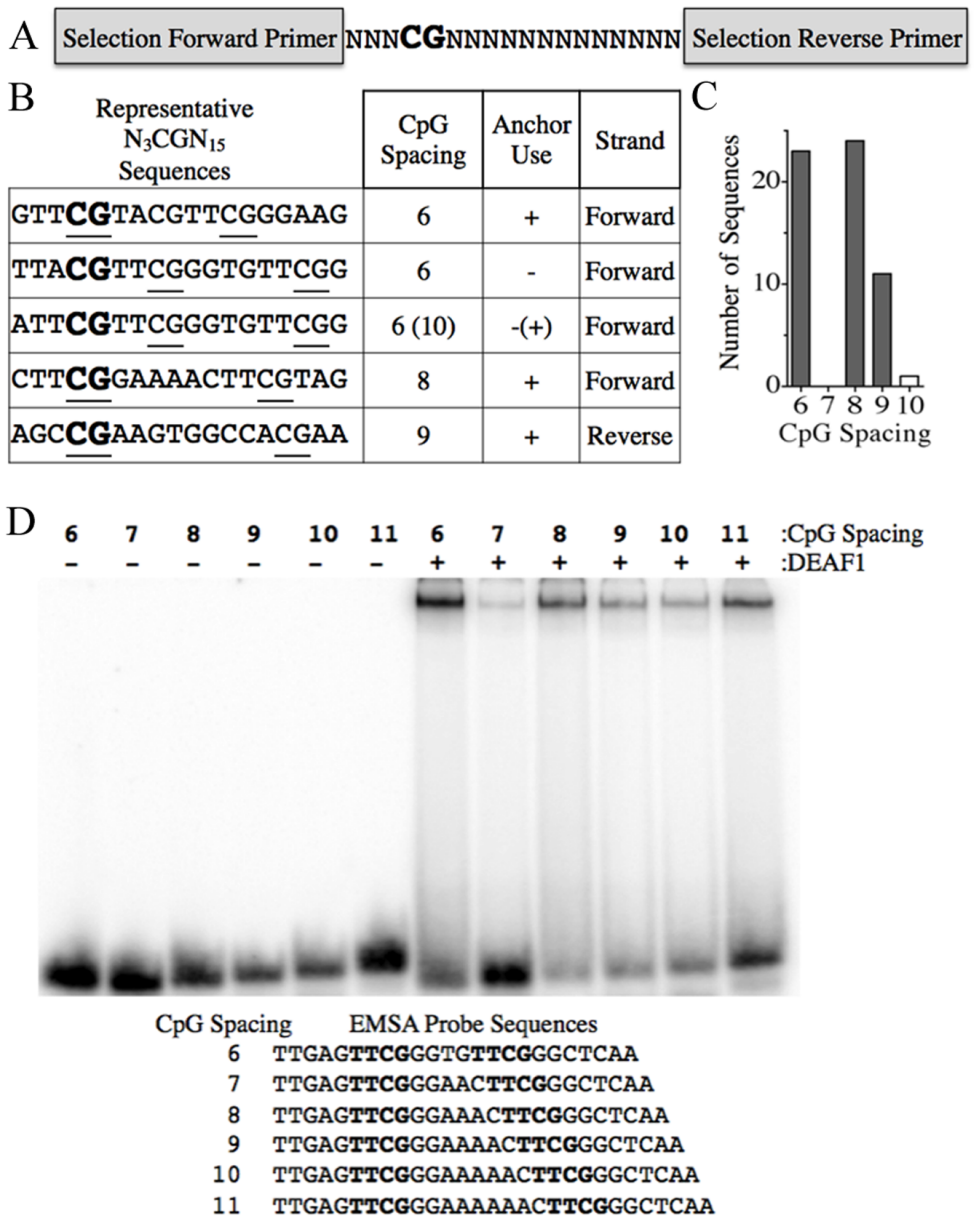
DEAF1 binds variably spaced half-sites. (A) Schematic diagram of the oligonucleotides in the 63-mer library used in the selection experiments. Nucleotide sequences of the primers are listed in the Materials and Methods. (B) Representative sequences for five of the 58 isolated sequences with indicated spacing, anchor use, and strand (+ or −). Bold nucleotides show location of the anchor CpG and underlined nucleotides show the CpG considered part of the DEAF1 binding half-sites. (C) Distribution of spacing between the CpG dinucleotides of the DEAF1 half-sites from the 58 selected sequences. Closed bars used the considered DEAF1 half-sites. Open bar indicates the oligonucleotide with 3 TTCG and a possible 10 nucleotide CpG spaced half-site. (D) EMSAs were performed using purified DEAF1-FLAG protein and the indicated ^32^P-labeled dsDNA probes with 6-11 CpG nucleotide spacing.

mouse#1-F 5′- AGAGTCTCTGAGGGTTTTCCTCGTGCCTG-3′ and

mouse#1-R 5′- CAGGCACGAGGAAAACCCTCAGAGACTCT -3′, that had been labeled with IR700 dye at the 5′ ends, HPLC purified (Integrated DNA technologies), and annealed together.

### Chromatin Immunoprecipitations (ChIP)

HEK293T cells (ATCC) were maintained in Dulbecco modified Eagle's medium (DMEM) supplemented with 10% fetal bovine serum (FBS) and penicillin/streptomycin and incubated in a humidified incubator at 37°C and 5% CO_2_. ChIP was performed using a modification of the Raghu Mirmira Lab protocol, University of Virginia. Briefly, cells on 100 mm plates were rinsed twice with cold 1x PBS and cross-linked with 1% formaldehyde for 10 min. 1 mL of 1.25 M glycine was added per 10 mL of 1% formaldehyde to stop the fixation. The cells were rinsed in PBS and collected. Cells were lysed in cold ChIP buffer (1% Triton X-100, 0.1% deoxycholate, 50 mM Tris pH 8.0, 150 mM NaCl, 5 mM EDTA) plus protease inhibitors (Roche Diagnostics) on ice for 30 min. Samples were sonicated using a Sonic Dismembrator and 1/8″ probe (Fisher Scientific) for 12 20-second pulses at 20% amplitude. ChIP was performed using 75–125 µg of protein. Samples were incubated at 4°C overnight with either 5 µL rabbit preimmune serum or 5 µL rabbit anti-DEAF1 [2]. Novex Protein G Dynabeads (Life Technologies) plus herring sperm DNA and BSA were added to each sample and were incubated at 4°C for an additional 2 hours. Samples were washed once each with: low salt (1% Triton X-100, 0.1% SDS, 2 mM EDTA, 20 mM Tris pH 8.0, 150 mM NaCl), high salt (1% Triton X-100, 0.1% deoxycholate, 50 mM Tris pH 8.0, 500 mM NaCl, 5 mM EDTA), and lithium chloride (0.25 M LiCl, 0.5% IGEPAL, 0.5% deoxycholate, 10 mM Tris pH 8.0, 1 mM EDTA). Protein-DNA complexes were eluted in 1% SDS, 0.1 M NaHCO_3_, and 0.1 mg/mL herring sperm DNA and then incubated at 65°C for 3–4 hours to reverse crosslinks followed by overnight EtOH precipitation. Following proteinase K digestion, DNA was isolated by phenol chloroform extraction and EtOH precipitation. The DNA was resuspended in water and used in PCR with the primer sets below.

ChIP Primers

EIF4G3 promoter forward 5′-ACCTCGCCTTTGGTCTTTC-3′

EIF4G3 promoter reverse 5′-AACGAGCAGAGCATCCAAC-3′

EIF4G3 Exon 2 forward 5′- TAGCCGGTGAAGGTAAAACG-3′

EIF4G3 Exon 2 reverse 5′-TAATCTGGGGACCTCACAGC-3′

## Results

### DEAF1 binds variably spaced half-sites

DEAF1 binds TTCG half-sites but the nucleotide requirements flanking the CpG dinucleotides and the spacing between those half-sites have not been defined. In order to determine optimal DEAF1 binding sites, a double stranded oligonucleotide pool was generated that contained an anchored CpG dinucleotide preceded by 3 and followed by 13 degenerate nucleotides (Fig. 1A). Selection experiments were performed using this pool with a combination of bacterial-expressed recombinant GST-DEAF1 and mammalian-expressed DEAF1-FLAG proteins (S1 Figure) to isolate DEAF1 target sequences (described in Materials and Methods). After six rounds of GST-DEAF1 affinity selection and one round of DEAF1-FLAG EMSA selection, 58 individual, non-redundant DEAF1 target DNA sequences were obtained (S2 Figure).

MEME analysis indicated that 43 of the 58 identified binding sequences utilized the anchor CpG dinucleotide as one of two CpG dinucleotides found in the DEAF1 binding motif. The other 15 binding sequences contained two CpG dinucleotides downstream of the anchor CpG (three CpG total). Based on the MEME analysis, the CpG-containing motifs downstream of the anchor CpG in these 15 sequences were considered as the preferred DEAF1 half-sites for alignment. Representative sequences are shown in Fig. 1B. Variable spacing between the identified DEAF1 half-sites was found with the second CpG-containing half-site occurring at 6 (N = 23), 8 (N = 24), or 9 (N = 11) nucleotides downstream of the first CpG half-site (Fig. 1C). One of the sequences containing three TTCG motifs is shown (Fig. 1B). Based on the half-site consensus analysis of this sequence, the 2^nd^ and 3^rd^ TTCG half-sites have a CpG spacing of 6 nucleotides, while the 1^st^ and 3^rd^ TTCG motifs have a CpG spacing of 10 nucleotides, suggesting either spacing could also contribute to binding. Also, in previous EMSA studies [3], [4] we had utilized a DNA sequence called N52-69 that is found in the *DEAF1* promoter region, is protected by DEAF1 in DNase protection assays [28], and contains an 11 nucleotide spacing between the two half-sites.

To confirm the ability of DEAF1 to bind variably spaced TTCG half-sites, dsDNA probes were generated that contained 2 TTCG motifs with 6, 7, 8, 9, 10 or 11 nucleotide spacing between the CpG dinucleotides (Fig. 1D). These probes were used in EMSA experiments with DEAF1-FLAG protein. DEAF1 was able to bind each of the probes, although, reduced binding was observed with the 7-space probe. Taken together, this indicates that DEAF1 can bind variably spaced CpG-containing half-sites.

### Nucleotides flanking the CpG dinucleotides of DEAF1 half-sites influence DNA binding

**Figure 2:**
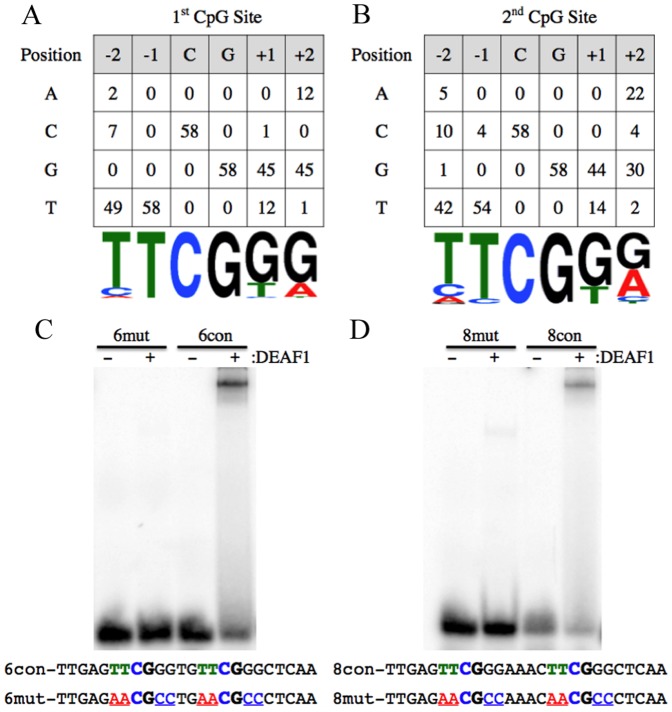
Nucleotides flanking the CpG dinucleotides of DEAF1 half-sites influence DNA binding. Nucleotide occurrence at the first (A) and second (B) DEAF1 half-sites. Consensus sequences are shown below the matrix for each half-site. EMSA were performed using the indicated 6-space (C) and 8-space (D) ^32^P-labeled dsDNA probes. Bold nucleotides indicate the half-site CpG dinucleotides. Underlined nucleotides are mutated in the 6mut and 8mut probes.

The first and second half-site sequences were then analyzed separately to determine the DEAF1 consensus binding sequence at each half-site. As shown in Figs. 2A and 2B both half-sites show high preference for TpT dinucleotides preceding the CpG. There was also a preference for GpG dinucleotides following the CpG but this preference appears to be less stringent in the second half-site. Mutation of the CpG dinucleotides in half-sites has previously been shown to eliminate DEAF1-DNA interactions [4] [3], but the influence of the nucleotides flanking either side of CpG on DEAF1-DNA interactions was not examined. To determine the importance of nucleotides preceding and following the CpG on DEAF1-DNA interactions, 6-space and 8-space dsDNA probes were generated that contained mutations in both the TpT and GpG dinucleotides flanking the CpG of both half-sites and used in EMSA. Compared to 6- and 8-space dsDNA probes containing two TTCGGG motifs, no DEAF1-DNA interactions were observed when the motifs were mutated to AACGCC (Figs. 2C and 2D).

### Target sequences with CpG methylation or single CpG dinucleotides show reduced binding to DEAF1

**Figure 3:**
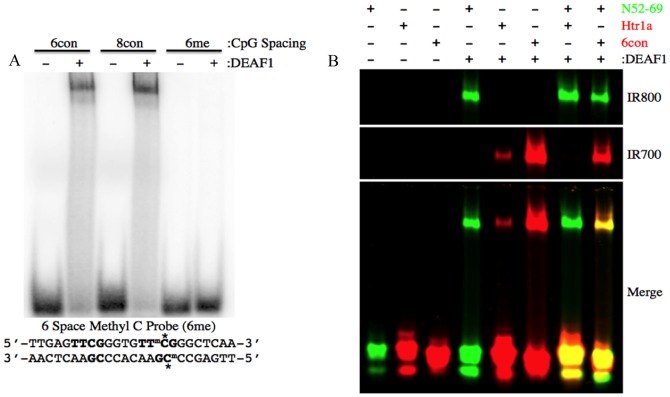
Target sequences with CpG methylation or single CpG dinucleotides show reduced binding to DEAF1. (A) EMSA was performed using ^32^P-labeled dsDNA probes with 6- and 8-space control probes (sequences in Fig. 2) and with 5-methylcytosine (^m^C*) bases in the second DEAF1 half-site. (B) EMSA was performed using equimolar amounts of N52-69 IR800, Htr1a IR700 and 6 space consensus (6con) IR700 probes.

Cytosine methylation most often occurs at CpG dinucleotides and can have a repressive effect on gene expression through multiple mechanisms, including negatively affecting direct transcription factor-DNA interactions [29], [30]. We sought to determine the effect of CpG methylation on DEAF1 interaction with a DEAF1 consensus-binding site. A 6-space dsDNA probe was generated that contained methylated cytosine nucleotides on both strands in the second CpG containing half-site. Compared to the unmethylated 6-space dsDNA probe, DEAF1 was unable to bind the methylated consensus sequence indicating that CpG methylation negatively affects DEAF1 binding (Fig. 3A).

It has been reported that DEAF1 binds a DNA sequence called “mouse#1” in the *Htr1a* promoter that contains a single CpG dinucleotide [27]. We confirmed DEAF1 binding to mouse#1 by EMSA, although binding was greatly reduced relative to probes with two TTCG half-sites (N52-69, 6con) and was eliminated in the presence of an equimolar amount of the N52-69 probe (Fig. 3B). The 6-spaced probe reduced DEAF1 binding to N52-69, suggesting it is also a preferred binding sequence relative to the 11-spaced N52-69 probe.

### DEAF1 binds to 8-spaced TTCG half-sites within the *EIF4G3* promoter

**Figure 4:**
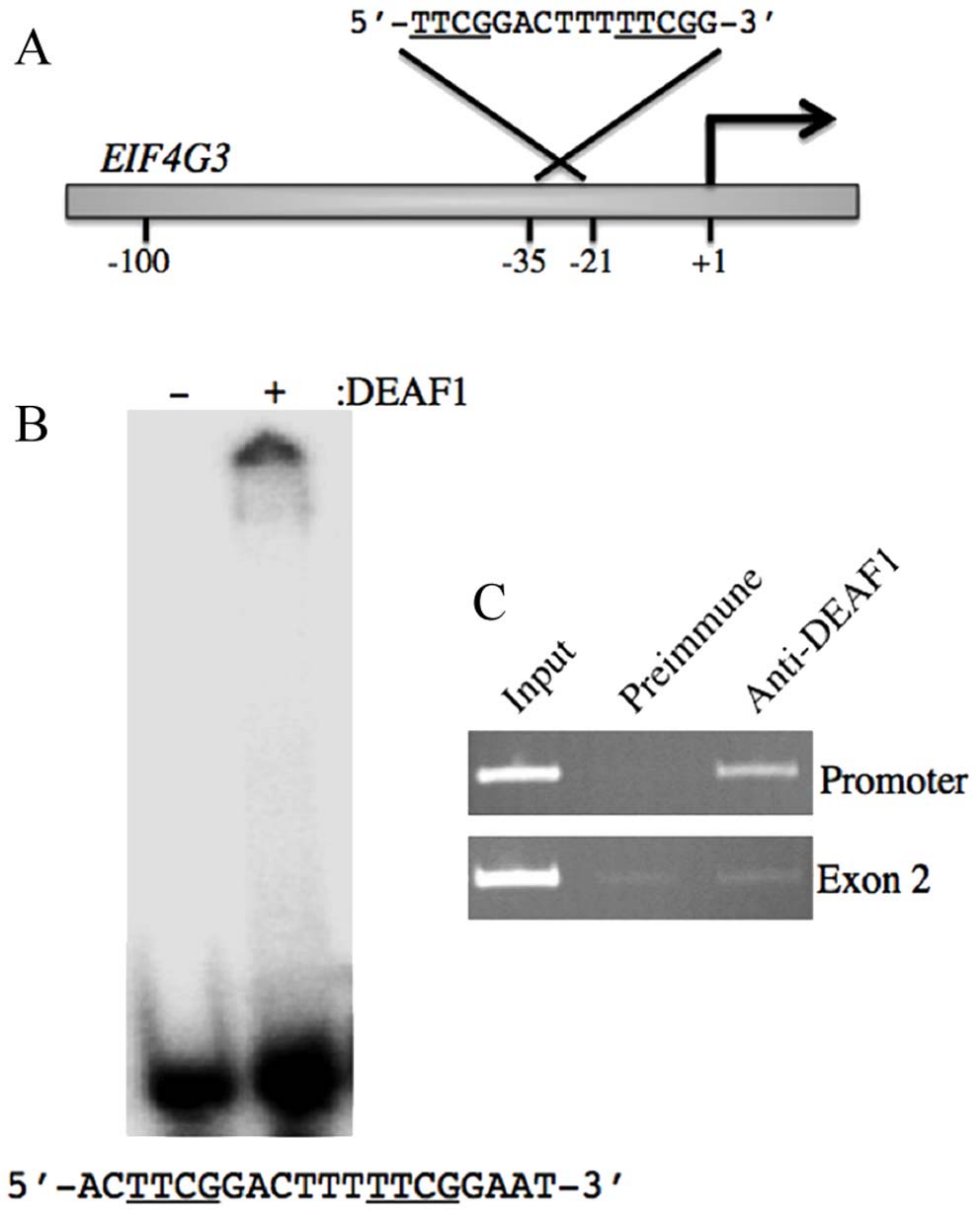
DEAF1 binds to 8-spaced TTCG half-sites within the human *EIF4G3* promoter. (A) Schematic of the *EIF4G3* promoter showing the location and the reverse complement sequence of the putative DEAF1 response element. Arrow indicates the transcriptional start site. (B) EMSA was performed using the indicated ^32^P-labeled dsDNA probe without (-) or with DEAF1 protein (+). (C) ChIP analysis of endogenous DEAF1 interaction with the *EIF4G3* promoter. ChIP PCR was performed using the indicated serum and primers that amplified either 249 bp of the promoter region or 298 bp of a region spanning exon 2.

Transcriptional promoter sequences in the human genome were scanned for potential DEAF1 response elements using the 8-space DEAF1 binding consensus sequence 5′-TTCGKNNNNNTTCGK-3′ using RSA-Tools Genomic Scale PatternSearch [26]. Over 200 genes were identified that contained this consensus sequence, including the human *EIF4G3* promoter (reverse complement shown in Fig. 4A). To determine the ability of DEAF1 to bind to the identified sequence within the *EIF4G3* promoter, a dsDNA probe that spanned the putative DEAF1 response element was generated and used in EMSA. As shown in Fig. 4B, DEAF1 was able to bind the *EIF4G3* promoter sequence. ChIP assays were then performed on HEK293T cells to evaluate the endogenous interaction of DEAF1 with the *EIF4G3* promoter. PCR primers were used that amplified either the DEAF1 consensus sequence-containing promoter region or a region 1527 bp downstream in exon 2 of the *EIF4G3* gene, which does not contain putative DEAF1 response elements. Anti-DEAF1 antibodies confirmed that DEAF1 was bound to the promoter region of *EIF4G3*, but was not bound to sequences in exon 2 compared to preimmune serum (Fig. 4C).

## Discussion

The half-site consensus sequence determined for DEAF1 binding in this study supports our previous finding [28] and further delineates spacing between the half-sites and adjacent nucleotides. Only DNA sequences with two CpG dinucleotide containing half-sites were identified and the spacing between half-sites was variable. To date, the ability of SAND domain transcription factors to bind variably spaced half-sites appears to be limited to DEAF1 and GMEB1/2. DEAF1 binds to TTCG half-sites with variable spacing of 6-11 nucleotides between the CpG dinucleotides, while GMEB1/2 transcription factors bind ACGT half-sites with 4-11 nucleotides between CpG dinucleotides [23]. Both DEAF1 and GMEB1/2 have a zinc-binding motif adjacent to the KDWK region as part of the SAND domain, although only the DEAF1 zinc-binding motif has been shown to affect DNA and/or protein interactions [4], [31]. Oligomerization of DEAF1, through DEAF1-DEAF1 protein interactions within the SAND domain [4] or coiled-coiled region [7], may facilitate the flexibility needed to bind variably spaced half-sites.

DEAF1 has been reported to bind a region within the human and mouse *Htr1a* (5HT_1A_ receptor) promoters and act as a transcriptional repressor in mouse serotonergic dorsal raphe neurons [15], [27]. The 26 bp sequence within the human promoter has a TTCG and an ACCGA with 10 nucleotides between the CpG dinucleotides and closely matches our determined binding consensus (Fig. 2A). However, the proposed DEAF1 binding site in the mouse *Htr1a* promoter (called mouse#1) only contains a single CpG dinucleotide [27]. This CpG occurs within the sequence CTCGTG and is preceded by a CTGAGGG (Fig. 2A) that lacks the seemingly critical CpG dinucleotide indicated by our derived binding consensus sequence. Interestingly, the monkey DEAF1 ortholog was first identified by interaction with a retinoic acid response element (RARE) that contains a single TTCG motif [2]. In addition, truncated recombinant HIS-tagged DEAF1 proteins encompassing the SAND domain (aa167-370 and aa167-306) can bind DNA sequences containing a single TTCG half-site [4]. The selection of oligonucleotide sequences with two or more TTCG half-sites and the effective competition of a single half-site with sequences with two half-sites (Fig. 3B) indicate that DEAF1 has lower affinity for single half-sites.

Transcriptional start sites and 5′ ends of transcripts of many housekeeping and tissue-restricted genes are enriched for CpG dinucleotides [32], thus the number of promoters that could be potentially regulated by DEAF1 because of its ability to bind variably spaced CpG dinucleotides is considerable. DEAF1 was shown to associate with approximately 200 sites on polytene chromosomes in *Drosophila* suggesting DEAF1 may act as a general transcription factor for hundreds of genes [22]. A DEAF1 consensus sequence, with 8-spaces between CpG dinucleotides, was identified in the human *EIF4G3* promoter and is conserved in the mouse *Eif4g3* promoter. We demonstrated that DEAF1 can bind to this specific DEAF1 consensus sequence within the *EIF4G3* promoter by EMSA (Fig. 4B) and that endogenous DEAF1 interacts with the *EIF4G3* promoter by ChIP (Fig. 4C). Mice deficient in DEAF1 showed decreased *Eif4g3* mRNA levels in pancreatic lymph nodes [5], and DEAF1 increased *Eif4g3* promoter activity [6] supporting DEAF1 as a transcriptional activator of *Eif4g3*.

Methylation of CpGs located within CpG islands of certain promoters is associated with transcriptional repression, mostly due to inability of transcription factors to bind to their consensus sequences [33]. CpG methylation has also been reported to occur as a consequence of gene repression due to chromatin condensation and probably stabilizes the heterochromatin structure [29]. CpG methylation can also produce DNA binding sites for specific transcription factors [34]. We found that CpG methylation of the second DEAF1 half-site markedly inhibited DEAF1-DNA interaction. Cytosine methylation at both CpG containing half-sites completely inhibits GMEB1/2-DNA interactions, while cytosine methylation at the second half-site only attenuated binding [23]. This may indicate that DEAF1 is more sensitive to methylated CpG half-sites compared to GMEB1/2.

The data presented here extends and broadens the understanding of the ideal DNA sequences that DEAF1 preferentially binds and these include CpG-containing half sites that are not methylated, variably spaced, and are influenced by surrounding nucleotides. However, as demonstrated by the mouse#1 sequence found in the *Htr1a* promoter, low affinity binding at other DNA sequences can occur and these may be relevant to DEAF1 transcriptional function. The results from this study should complement and help guide future studies that address localization of DEAF1 to endogenous chromosomal sites in appropriate target tissues.

## Supporting Information

S1 Figure **Relative purities of GST-DEAF1 and DEAF1-FLAG. GST-DEAF1 and DEAF1-FLAG proteins (500 ng) were separated by SDS-PAGE and the gel was stained with Coomassie blue. Molecular weight standards are also shown.**
(TIF)

S2 Figure **Nucleotide sequences of the oligonucleotides selected by DEAF1 binding.**
(TIF)
