# Supplementary figures and images for: DEAF1 Binds Unmethylated and Variably Spaced CpG Dinucleotide Motifs
Source: PLoS One. 2014 Dec 22;9(12):e115908. doi: 10.1371/journal.pone.0115908 (PMC4274154; doi:10.1371/journal.pone.0115908)

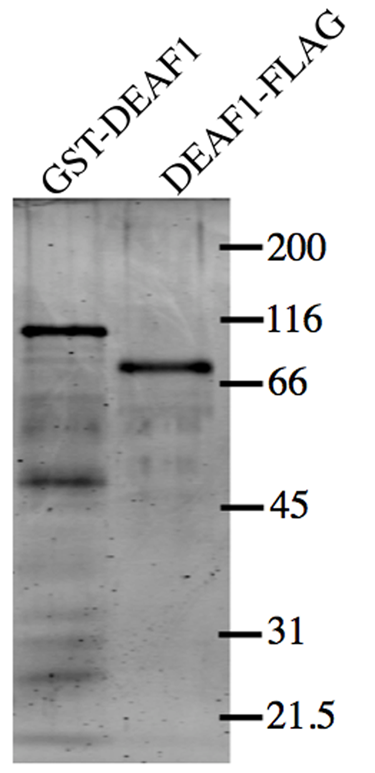

Supplement: S1 Figure — Relative purities of GST-DEAF1 and DEAF1-FLAG. GST-DEAF1 and DEAF1-FLAG proteins (500 ng) were separated by SDS-PAGE and the gel was stained with Coomassie blue. Molecular weight standards are also shown. (TIF) [file pone.0115908.s001.tif]

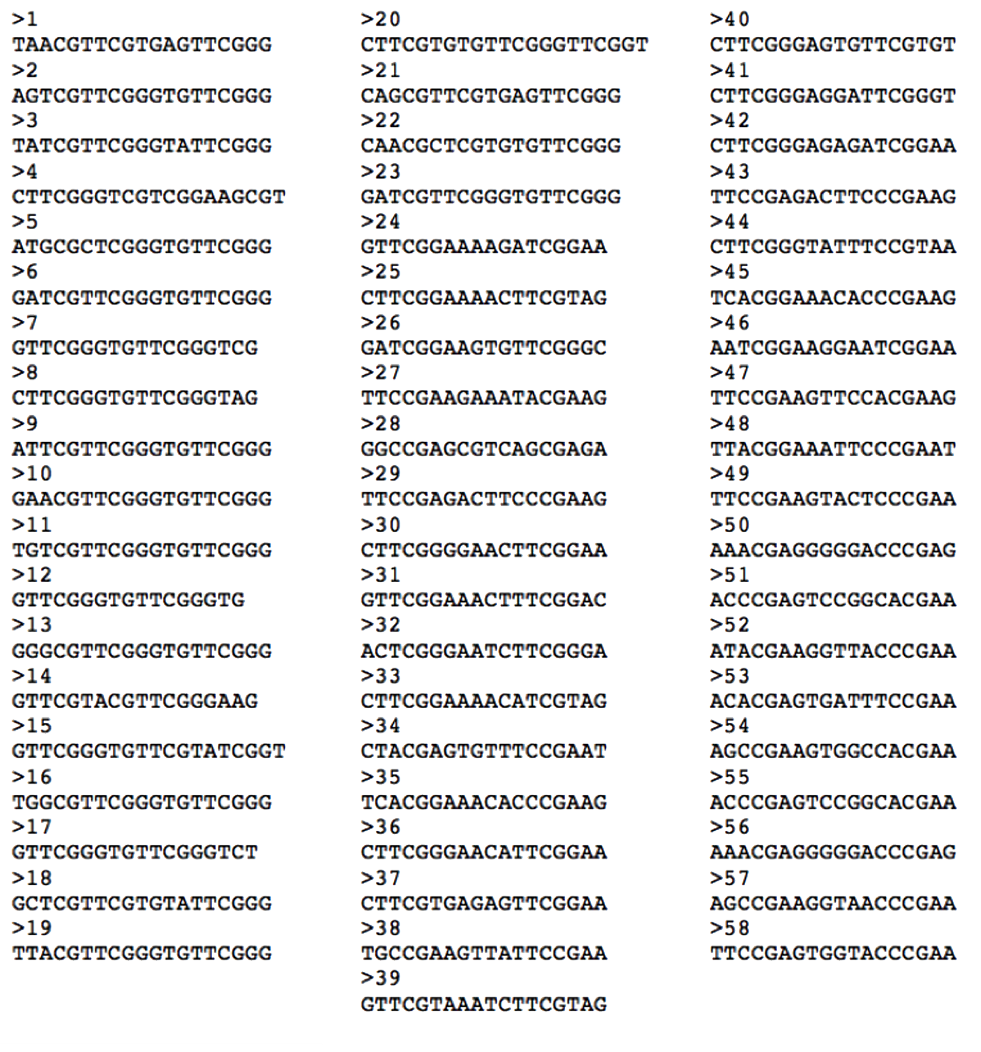

Supplement: S2 Figure — Nucleotide sequences of the oligonucleotides selected by DEAF1 binding. (TIF) [file pone.0115908.s002.tif]
